# Peer Review of “Use of a Specialist Telephone Consultation Line for Long COVID in Primary Care in British Columbia: Retrospective Descriptive Quality Improvement Study”

**DOI:** 10.2196/89735

**Published:** 2026-02-10

**Authors:** Saidi Olayinka Olalere

**Affiliations:** 1 Georgia Southern University, Statesboro, GA, United States

**Keywords:** internal medicine, long COVID, COVID-19, SARS-CoV-2, GP, general practice, general practitioner, consult, respiratory, infectious, respiration, primary care, telephone, telehealth

*This is the peer-review report for “Use of a Specialist Telephone Consultation Line for Long COVID in Primary Care in British Columbia: Retrospective Descriptive Quality Improvement Study.”*

## Round 1 Review

### General Comments

This paper [1] is structured well, with an analysis of the data obtained. The authors need to present how this data will assist the province with some figures or data.

### Specific Comments

#### Major Comments

1. The authors mention that 6 calls were excluded but never gave an analysis of the trend of the calls.
2. Can the 6 calls drive some conclusions that can assist with the paper?
3. Can the authors give a trend line for the period of these calls and if there are related cases of different calls?
